# A Neurodevelopmental Model of Eating Disorder Risk in Midlife and Older Adulthood

**DOI:** 10.1093/geroni/igaf122.3041

**Published:** 2025-12-31

**Authors:** Hannah Heintz-Monette, Ann Haynos, Kelsey Hagan, Laura Berner

**Affiliations:** Virginia Commonwealth University, Richmond, Virginia, United States; Virginia Commonwealth University, Richmond, Virginia, United States; Virginia Commonwealth University School of Medicine, Richmond, Virginia, United States; Icahn School of Medicine at Mount Sinai, New York, New York, United States

## Abstract

Prior research has identified the interaction of rapid neurocognitive changes with psychosocial stressors as a major precipitant of disordered eating in adolescence. However, there has been little discussion of how similar processes may set the stage for disordered eating in later life. Here, we present a novel framework for the promotion and maintenance of eating pathology in midlife and beyond. First, we review the critical neurobiological alterations that occur during this unique developmental stage and connect these alterations to established risk factors for disordered eating in adolescence. Second, we discuss how unique, aging-related psychosocial stressors interact with brain aging processes to increase vulnerability. We then highlight how our framework sheds light on potential new targets for psychopharmacology and psychotherapy in older adults experiencing disordered eating. Based on this framework, we outline the following future research directions: 1) directly investigate the influence of hormonal, neurocognitive, neurobiological, and environmental aging changes on eating disorder behavior; 2) distinguish pathways from aging to eating pathology that differ as a function of aspects of identity or specific developmental period; 3) establish shared and distinct mechanisms influencing the emergence or worsening of different eating disorder diagnoses and presentations in later life; and 4) identify potential targets for prevention and treatment of eating disorders as vulnerable individuals age. Finally, we advocate for enhanced awareness and screening for eating pathology in midlife and older adults, as well as for a multidisciplinary approach to the treatment of eating disorders in this population.

